# Repeated bouts of load carriage alter indirect markers of exercise‐induced muscle damage, liver enzymes, and oxygen‐carrying capacity in male soldiers

**DOI:** 10.14814/phy2.70268

**Published:** 2025-07-30

**Authors:** Chad R. Straight, Kari L. McKenzie, Ava L. Sargent, Kenneth Racicot, Adrienne Hatch‐McChesney, Tshinanne V. Ndou, Kevin S. O'Fallon

**Affiliations:** ^1^ Combat Capabilities Development Command Soldier Center Natick Massachusetts USA; ^2^ Oak Ridge Institute for Science and Education Oak Ridge Tennessee USA; ^3^ United States Army Research Institute of Environmental Medicine Natick Massachusetts USA

**Keywords:** immune cells, inflammation, military, performance, skeletal muscle

## Abstract

Soldiers are often required to carry heavy external loads over multiple days, which may degrade physical performance. We investigated the effects of repeated load carriage bouts on indirect markers of exercise‐induced muscle damage, liver enzymes, and oxygen‐carrying capacity in active‐duty infantrymen. Fourteen male soldiers (age = 24.6 ± 1.1 y; BMI = 25.7 ± 0.7 kg/m^2^) underwent a 5‐day protocol, consisting of baseline/familiarization, 3 load carriage bouts, and a recovery day. There were reductions in maximal voluntary contraction strength (*p* < 0.05), with the knee flexors and trunk extensors showing the greatest declines. Each load carriage bout produced an inflammatory response, including increases in leukocyte subtypes (neutrophils and monocytes) and monocyte chemoattractant protein‐1 (*p* < 0.05). At the end of the protocol, serum liver enzymes were elevated, and erythrocytes and hematocrit were lower than baseline (*p* < 0.05). In addition, greater circulating leukocytes at baseline predicted lower knee and trunk torque during recovery. Repeated bouts of load carriage reduce muscle strength and cause inflammation consistent with exercise‐induced muscle damage, alter liver function tests, and decrease oxygen‐carrying capacity in male soldiers, which could compromise readiness for prolonged and/or intense military operations.

## 1 INTRODUCTION

Prolonged and/or repeated bouts of extreme physical exertion are an inevitable aspect of military training and operations, particularly among infantry personnel. A primary example of this is load carriage, an activity where an external load‐bearing apparatus is placed upon the thoracic cavity (Faghy et al., 2022). In the U.S. Army, infantry soldiers can be required to carry loads ranging from 30% to 70% of body mass (Department of the Army, 2022), often over consecutive days and variable terrain. A single bout of loaded walking can alter gait kinematics (Walsh & Low, 2021), energy expenditure (Huang & Kuo, 2014), and markers of bone resorption and formation (Staab et al., 2023). However, other physiological effects of load carriage remain poorly understood, such as those on the immune system and skeletal muscle function. As load carriage is an essential military task, understanding how it may impact physical performance (Boffey et al., 2019) and injury risk (Gill et al., 2023) is a critical step toward advancing soldier health and readiness.

Repeated bouts of heavy load carriage could have a detrimental impact on soldier physical performance by causing exercise‐induced muscle damage (EIMD), which is indicated by skeletal muscle weakness, systemic inflammation, an efflux of intramyocellular proteins into the blood, and delayed‐onset muscle soreness (Clarkson & Hubal, 2002). Of these signs, skeletal muscle function has been identified as the best indirect marker of EIMD (Damas et al., 2016) and is a critical determinant of the ability to complete common military tasks (Nindl et al., 2015). A couple of studies (Blacker et al., 2010, 2013) found that 2 h of loaded walking reduced knee extensor torque by ~15%, with another reporting that a second bout exacerbated the loss of strength (James et al., 2021), but those studies involved recreationally active young adults. Arduous field exercises can also decrease strength (Hamarsland et al., 2018; Vikmoen et al., 2024), but have included additional stressors (e.g., energy restriction and sleep deprivation) and/or resulted in weight loss, meaning reduced muscle function could be due to factors beyond EIMD. Thus, whether active‐duty soldiers experience a similar magnitude of strength loss due to repeated load carriage bouts is unclear.

Another hallmark of EIMD is systemic inflammation (Paulsen et al., 2012). If load carriage does indeed lead to mechanical tissue damage, then an acute inflammatory response consistent with EIMD would be expected. Surprisingly, only two studies have reported the effects of load carriage on a single cytokine (Jensen et al., 2019; Pasiakos et al., 2016) and none have examined changes in immune cell counts. Mechanically injured skeletal muscle produces an acute inflammatory response coordinated by the innate immune system, including neutrophils and monocytes, and chemokines regulate the migration of these cells to peripheral sites of tissue damage. A key chemokine responsible for myeloid cell recruitment is monocyte chemoattractant protein‐1 (MCP‐1) (Gschwandtner et al., 2019; Takahashi et al., 2009). Preclinical work describes a role for MCP‐1 in tissue repair and remodeling (Lu et al., 2011), but the effects of exercise on circulating MCP‐1 levels have been equivocal, with studies reporting either an increase (Paulsen et al., 2005) or decrease (Ihalainen et al., 2014; Lagzdina et al., 2023), which may reflect the presence and/or extent of EIMD. For these reasons, the systemic inflammatory response to load carriage is best understood through the activity of both immune cells and signaling molecules (e.g., chemokines) with established roles in tissue damage.

Load carriage imposes unique physiological demands and may have systemic effects that extend beyond traditional indicators of EIMD. For example, an acute bout of unaccustomed resistance training elevates liver enzymes in serum (Pettersson et al., 2008) and the mechanical strain placed upon muscle fibers during heavy load carriage could produce a similar response. Repeated bouts of load carriage could also affect physical performance by altering red blood cell parameters. Prolonged endurance events, such as a marathon (Kratz et al., 2002), reduce erythrocyte and hematocrit levels via exercise‐induced hemolysis, and this translates to a loss of blood (Elzik et al., 2006), which may impair oxygen‐carrying capacity and subsequent performance. Similar changes could occur during load carriage, especially given the heavy external loads that may amplify compressive forces on muscle capillaries during foot strike (Telford et al., 2003), but this remains unknown.

In this context, we investigated the impact of repeated load carriage bouts on indirect markers of EIMD, liver function tests, and oxygen‐carrying capacity in male soldiers. Based on previous studies reporting changes in muscle strength (Blacker et al., 2010, 2013; James et al., 2021) and cytokine responses (Jensen et al., 2019; Pasiakos et al., 2016) after 1–2 bout(s) of load carriage, we hypothesized that our protocol would result in a cumulative loss of knee and trunk strength, systemic inflammation, elevated enzyme levels, and soreness in multiple body regions, all of which would suggest compromised physical readiness.

## 2 MATERIALS AND METHODS

### 2.1 Participants

Participants were healthy active‐duty U.S. Army infantrymen aged 18–39 y to be consistent with the demographics of the military (Department of Defense, 2021). Men and women were both recruited during briefing sessions; however, only male soldiers participated. Twenty‐one individuals were enrolled for participation, and 14 completed the study. Seven participants did not complete the protocol for the following reasons: exacerbation of unreported musculoskeletal injuries (*n* = 5), unrelated illness (*n* = 1), and a personal matter (*n* = 1). To be eligible for participation, individuals were required to meet the American College of Sports Medicine (ACSM) (Garber et al., 2011) physical activity guidelines on cardiorespiratory exercise (moderate‐intensity activity for 30 min on 5 d/week or vigorous‐intensity activity performed for 20 min on 3 d/week). In addition, eligible individuals met the following inclusion criteria: experience carrying loads ≥30% of their body mass on at least three separate occasions, with each session lasting ≥30 min in the last 12 months; infantry military occupational specialty (MOS); completed basic training and a standard live fire marksmanship qualification; and were willing to comply with diet and exercise restrictions of the study. Individuals were excluded from participation for any of the following reasons: history of neck or lower back problems (including herniated intervertebral disks); orthopedic injuries limiting range of motion at the shoulder, hip, knee, or ankle; cardiopulmonary conditions that could be exacerbated by vigorous‐intensity physical activity (e.g., exercise‐induced asthma); neurologic disorders including traumatic brain injury or stroke; movement or motor control disorders (e.g., paralysis, spasticity of muscles, muscle tremors, or dystonia); uncorrected vision problems; unwillingness to abstain from alcohol use 24 h prior to data collection; and unwillingness to abstain from caffeine or nicotine use 2 h prior to testing each day. All individuals provided written informed consent prior to participation, and all procedures within this protocol were approved by the Institutional Review Board at the U.S. Army Combat Capabilities Development Command Armaments Center (Picatinny, NJ, United States). Data collection was performed at the U.S. Army Combat Capabilities Development Command Soldier Center (Natick, MA, United States).

### 2.2 Experimental protocol

This protocol used a repeated measures design to investigate the effects of multiple load carriage bouts on indirect markers of EIMD, liver enzymes, and oxygen‐carrying capacity in soldiers eating a controlled diet. Each participant completed 5 consecutive days of data collection: a baseline day, three bouts of load carriage exercise (BOUT1, BOUT2, and BOUT3), and a recovery day (REC).

Participants arrived at the laboratory each morning after a 10‐h overnight fast. For 24 h prior to each visit, individuals were instructed to refrain from strenuous physical activity (outside of the laboratory) and alcohol consumption. During the first visit, all study procedures were reviewed with participants. Then, a fasted blood sample was collected, followed by the distribution of daily meals based on individualized diet prescriptions and self‐administered questionnaires to assess demographics, muscle soreness, and other lifestyle behaviors (sleep, physical activity, and diet). Height was measured with an anthropometer (GPM, Bitziberg, Switzerland) and body weight with a digital scale (Seca 869; Seca, Hamburg, Germany) while the participant was wearing lightweight clothing (e.g., compression shirt and shorts), combat boots, and an unloaded weight vest. Skinfold measurements were performed using a Lange skinfold caliper (Beta Technology, Santa Cruz, CA) at 7 sites (abdominal, triceps, chest, midaxillary, subscapular, suprailiac, and thigh) to estimate percent body fat in accordance with ACSM guidelines (Jackson & Pollock, 1985). Participants were familiarized with isokinetic dynamometry, load carriage tasks, and all other testing procedures presented herein, as well as other study measures (gait mechanics) that have been reported elsewhere (Brink et al., 2024). Lastly, prior to data collection, participants were instructed to refrain from using all medications (nonsteroidal anti‐inflammatory drugs; NSAIDs), topical analgesics, massage, and hot or cold showers, baths, or compresses known to interfere with study outcomes (e.g., muscle soreness) for the duration of the protocol.

**FIGURE 1:**
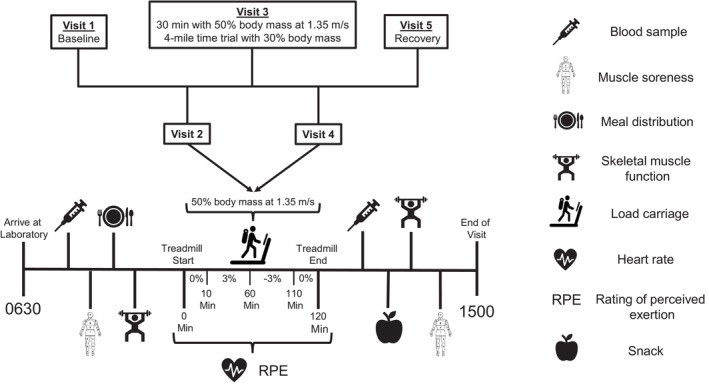
Overview of the experimental protocol. During each day of the study, participants arrived to the laboratory at 0630 following a ~10‐h overnight fast. Visit 1 included familiarization with the protocol and anthropometric measurements (height, weight, and body composition). Before and after each bout of load carriage exercise (Visits 2–4), a venous blood sample was collected, knee and back/trunk muscle function was measured via isokinetic dynamometry, and perceived muscle soreness was reported. Individualized diet prescriptions were distributed in the morning, and a snack was provided in the afternoon. On BOUT1 (Visit 2) and BOUT3 (Visit 4) of the protocol, participants completed 2 h of loaded walking (50% body mass) at 1.35 m/s and variable grade, including 0% (0–10 min), 3% (10–60 min), −3% (60–110 min), and 0% (110–120 min). On BOUT2, 30 min of loaded walking (50% body mass) was followed by a 4‐mile time trial with reduced load (30% body mass). During each load carriage bout, exercise intensity was monitored via continuous heart rate measurements and rating of perceived exertion was reported every 10 min. During Visit 5, all primary outcomes were measured to characterize recovery following the protocol.

After the baseline/familiarization session on Visit 1, participants completed one bout of load carriage exercise during each of the next three visits (BOUT1, BOUT2, and BOUT3). All primary outcomes (skeletal muscle function, blood, and soreness) were measured daily, including familiarization on the baseline day (Visit 1), before and after each load carriage bout (Visits 2–4), and on the recovery day (Visit 5). An overview of the experimental protocol, including primary outcomes and procedures during the load carriage days (Visits 2–4), is shown in Figure 1.

### 2.3 Load carriage exercise

During BOUT1 (Visit 2) and BOUT3 (Visit 4), participants performed 2 h of walking on an instrumented treadmill (Integrated Force Plate Treadmill, Advanced Mechanical Technology, Inc., Watertown, MA) at 1.35 m/s (3.0 mph), a gait speed recommended in Army doctrine (Department of the Army, 2022) and consistent with previous load carriage studies (Loverro et al., 2019; Schiffman et al., 2009), resulting in 9.7 km (6.0 miles) of distance covered. During the trials, participants donned a weight vest equivalent to 50% of body mass, using a weight distribution (40% anterior and 60% posterior) that aligned with typical configurations in field training and combat scenarios (Grenier et al., 2012). For the first 10 min, participants walked at a 0% grade to determine a baseline walking pattern, as previously reported (Brink et al., 2024). After 10 min, participants transitioned to a 3% incline at the same velocity and maintained this grade and speed until the halfway mark of the task (1 h), upon which the treadmill grade was adjusted to a 3% decline for the next 50 min, and finally back to a 0% grade for the final 10 min. Exercise intensity was monitored continuously using a Polar® (Kempele, Finland) chest‐borne heart rate (HR) monitor. Subjective measures were also obtained every 10 min throughout the task using the Borg Rating of Perceived Exertion (RPE) scale (Borg, 1982). Participants were allowed self‐selected 5‐min rest periods (≤3) during the task to maximize safety and limit attrition due to the vigorous nature of the activity.

During BOUT2 (Visit 3), participants performed 30 min of treadmill walking under the same conditions (walking at 1.35 m/s with 50% body mass) as the other load carriage bouts. During this task, participants walked for 10 min at 0% grade, followed by 10 min at a 3% incline and 10 min at a 3% decline. After the 30‐min walk, participants quickly (≤5 min) transitioned to a different treadmill (4Front, Woodway USA, Inc., Waukesha, WI) and load was reduced to 30% of body mass (distributed symmetrically) to represent the load typically carried during accelerated movement‐to‐contact scenarios (Department of the Army, 2022). At this point, participants completed a 4‐mile time trial (6.4 km), whereby pace was self‐selected, and the objective was to cover the distance as quickly as possible. During this portion of the task, the treadmill was adjusted to a 1% incline, as this grade has been shown to best simulate outdoor running (Jones & Doust, 1996). Gait speed was held constant at 1.35 m/s for the first 5 min of the trial, after which participants were able to adjust treadmill speed in a blinded manner. Participants were asked to report RPE upon completing each 0.5‐mile increment throughout the time trial.

### 2.4 Dietary prescription

Study diets were implemented to ensure that participants subsisted exclusively on military rations as they typically do during field training and combat scenarios, and water was allowed ad libitum. Registered dietitians developed individualized menus for each participant using the Combat Rations Database (U.S. Army Combat Capabilities Development Command and U.S. Army Research Institute of Environmental Medicine) and Food Processor SQL (version 10.14, ESHA Research, Salem, OR). Menus were derived from components of U.S. military Meals Ready‐to‐Eat (MRE; menu 40 Case A; Ameriqual, Evansville, IN) and First Strike Rations (FSR; 2020; Sopakco, Mullins, SC). Trained research staff packed and provided all foods and beverages, as well as a menu log for recording the time each item was consumed, on the morning of each day. Participants were instructed to consume (1) only foods and beverages provided by study staff each day, and (2) all provided items before the end of each day (e.g., 1900), with no restrictions on meal timing. All trash/wrappers and unconsumed or partially consumed items were returned the following day, along with the menu log, and reviewed by study staff. Daily energy intake and macronutrient composition were calculated based on the percentage (0%, 25%, 50%, and 100%) of each ration item consumed. All participants complied with study diets according to daily physical inventory and in‐person review of menu logs with study staff.

### 2.5 Skeletal muscle function

Maximal voluntary contraction (MVC) of the knee and trunk extensors and flexors was measured via a dynamometer (System 4 Pro, Biodex Medical Systems, Shirley, NY) under both isometric and isokinetic conditions. For each test, participants performed three warm‐up repetitions at submaximal efforts (25%, 50%, and 75%) immediately prior to the MVC trials. For the knee joint, isometric MVC was measured on the dominant leg at a knee angle of 60° flexion. Participants performed three repetitions, with each separated by a 30‐s rest period, and the peak torque was used for analysis. After a 2‐min rest period and practice trials, concentric isokinetic MVC of the knee extensors and flexors was measured during 5 repetitions at 60°/s, and, after an additional 2‐min rest period, during 20 repetitions performed at 120°/s. Following the knee tests, isometric MVC of the trunk musculature was measured at 90° flexion, with 30‐s rest periods between repetitions, and the peak torque of three repetitions was used for analysis. Concentric isokinetic MVC of the trunk extensors and flexors was measured under the same conditions (five repetitions at 60°/s and 20 repetitions at 120°/s) and using the same rest periods and practice trials.

### 2.6 Blood sampling

Blood samples were collected from an antecubital vein in the morning after a 10‐h overnight fast, and participants had rested for ~15 min, and again immediately following each load carriage task. Samples were drawn into 6‐ml K2 EDTA and serum‐separation vacutainers (Beckton Dickinson, Franklin Lakes, NJ) for plasma and serum, respectively. K2 EDTA samples were stored on ice and centrifuged at 3000*g* at 4°C for 10 min, and serum samples were allowed to clot at room temperature for 30 min, followed by centrifugation at 12,000*g* for 10 min. All samples were divided into 2‐mL aliquots and stored at −80°C until analysis. A complete blood count with differential was measured in whole blood with a hematology analyzer (CELL‐DYN Ruby, Abbott, Abott Park, IL) to examine circulating levels of leukocytes and subfractions (neutrophils, monocytes, lymphocytes, basophils, and eosinophils), erythrocytes (hemoglobin and hematocrit), and platelets. A comprehensive metabolic panel was performed on serum with a blood chemistry analyzer (Piccolo Xpress, Abaxis Inc., Union City, CA) for concentrations of hepatic enzymes, including alanine transaminase, alkaline phosphatase (ALP), and aspartate aminotransferase (AST). Serum creatine kinase activity was measured using a colorimetric assay kit (catalog # MAK116; Sigma Aldrich, St. Louis, MO) in which a coupled enzymatic reaction that produces nicotinamide adenine dinucleotide phosphate (NADPH) is proportionate to the creatine kinase activity in the sample. NADPH absorbance of 340 nm was measured on a plate reader (BMG Labtech, Cary, NC), from which creatine kinase activity was calculated per manufacturer's instructions. Lastly, monocyte chemoattractant protein‐1 (MCP‐1) was measured given its critical role in myeloid cell chemotaxis (Deshmane et al., 2009). Serum samples were analyzed using the Human ProcartaPlex Mix & Match 7‐plex kit (catalog # PPX‐07; Thermo Fisher Scientific Inc., Waltham, MA) on the Luminex MAGPIX system with xPONENT 4.2 (Luminex Corporation, Austin, TX). The assay was performed per manufacturer's instructions, and concentration was calculated from a 5‐parameter logistic standard curve.

### 2.7 Muscle soreness

Perceived muscle soreness was assessed using the Rating of Pain, Soreness, and Discomfort (RPSD) questionnaire (Corlett & Bishop, 1976). The RPSD questionnaire consisted of a visual anatomical diagram with letter designations indicating specific body segments of the anterior and posterior chains. Participants were instructed to rate perceived muscle soreness in each segment on the diagram using a 5‐point scale (none, slight, moderate, severe, or extreme).

### 2.8 Statistical analysis

Skeletal muscle function has been identified as the best indirect marker of EIMD (Damas et al., 2016); thus, our sample size estimate was based on the anticipated change in joint torque production following multiple bouts of load carriage exercise. A previous study (Blacker et al., 2013) reported a ~15% reduction in MVC of the knee extensors after a single bout of load carriage in 10 participants (within‐group Cohen's *d* = 0.80). Our study used a repeated measures design, which increases statistical power beyond paired samples, however we adopted a more conservative approach and calculated power based on a medium effect. We determined that a sample of 9 participants would provide 90% power to detect a medium effect (partial eta squared = 0.06) of load carriage exercise on isometric knee extensor MVC, assuming *α* = 0.05 and a correlation between repeated measurements of 0.80.

All data were screened for normality using Shapiro–Wilk tests, distribution statistics (skewness and kurtosis), and visual inspection of histograms and box plots. A mixed model one‐way repeated measures analysis of variance (ANOVA) was used to examine the main effect of time on all primary outcomes. When a significant main effect of time was observed, model effect sizes were calculated using eta squared (*η* ^2^) and interpreted as small (≥0.01), medium (≥0.06), or large (≥0.14) (Cohen, 1988). For significant main effects, pairwise comparisons were analyzed with Tukey's honest significant difference test and effect sizes (Cohen's *d*) were interpreted as small (*d* = 0.20), medium (*d* = 0.50), or large (*d* = 0.80) (Cohen, 1988). Friedman's nonparametric one‐way ANOVA by ranks was used to examine the effect of load carriage on ratings of perceived muscle soreness. The Dunn method with Bonferroni adjustments for multiple comparisons was performed if significant differences in ranks were detected. Linear regression was used to examine relationships between outcomes of interest, including muscle and liver enzymes, as well as circulating leukocyte levels and knee/trunk torque at baseline and recovery. All analyses were conducted using JMP (v15) and SPSS for Windows (version 29). Data are presented as mean ± SE, and statistical significance was set at *p* < 0.05.

## 3 RESULTS

### 3.1 Participant characteristics

Descriptive characteristics are shown in Table 1. Compared to those participants who completed the study, non‐completers (*n* = 7) were heavier (96.9 ± 4.9 vs. 82.9 ± 2.7 kg; *p* = 0.0145), with a greater body mass index (29.7 ± 0.8 vs. 25.7 ± 0.7 kg/m^2^; *p* = 0.0027) and percent body fat (18.0 ± 1.7 vs. 10.3 ± 0.8%; *p* = 0.0003), and had lower Army Combat Fitness Test scores (392.6 ± 63.3 vs. 528.6 ± 20.3 points; *p* = 0.0177). Among the 14 participants who completed the study, body weight remained stable throughout the protocol (−0.3 ± 0.3 kg; *p* = 0.4841).

**TABLE 1:** Participant characteristics (*n* = 14).

	Mean ± SE	Min‐max
Age (y)	24.6 ± 1.1	19–33
Height (m)	1.8 ± 0.1	1.7–1.9
Body mass (kg)	82.9 ± 2.7	64.1–100.2
Body mass index (kg/m^2^)	25.7 ± 0.7	21.5–31.4
Percent body fat (%)	10.3 ± 0.8	5.1–14.1
ACFT Score (points)^a^	528.6 ± 20.3	283–597
Two‐mile run time (min:sec)	13:58 ± 1:29	11:16–17:54
Service time (y)	3.8 ± 0.6	1.0–8.0

Abbreviation: ACFT, army combat fitness test.

^a^ Point values indicate aggregate scores across six fitness categories toward the maximum possible score of 600.

### 3.2 Load carriage exercise

The participants included in this analysis completed all bouts of load carriage. In general, loaded walking (50% of body weight) corresponded to moderate‐intensity aerobic exercise (Garber et al., 2011), based on percent of maximal HR (BOUT1 = 64.9 ± 1.6%; BOUT2 = 61.2 ± 1.7%; BOUT3 = 64.5 ± 1.5%) and RPE (BOUT1 = 13.8 ± 0.5; BOUT2 = 12.5 ± 0.7; BOUT3 = 14.0 ± 0.6). On BOUT2, the loaded walk was followed by a 4‐mile time trial with 30% of body weight (completion time: 52:08 ± 1:30 min), which elicited a greater percent of maximal HR (81.6 ± 2.5%) and RPE (17.4 ± 0.7) and aligned with vigorous‐intensity aerobic exercise (Garber et al., 2011).

### 3.3 Skeletal muscle function

**FIGURE 2:**
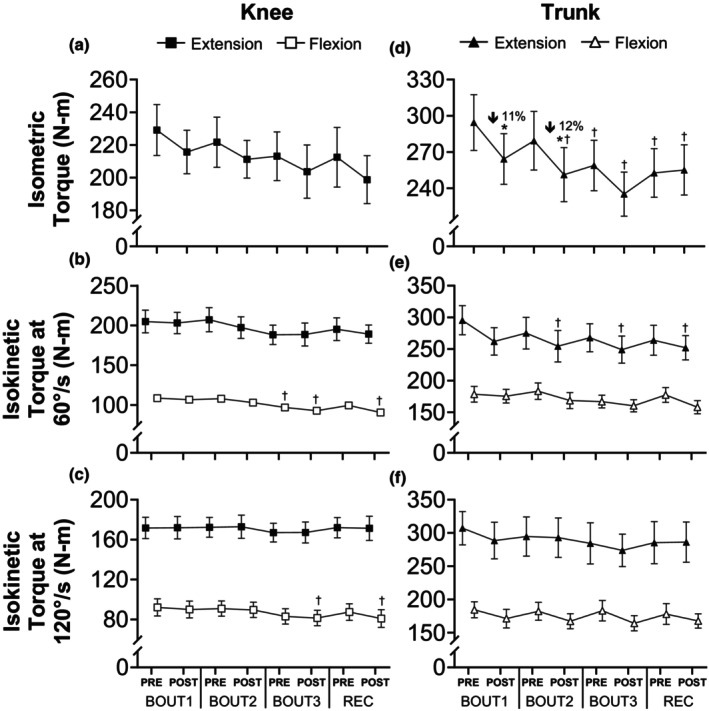
Skeletal muscle function in response to repeated bouts of load carriage exercise. Isometric and isokinetic knee (a–c) and trunk (d–f) flexion and extension torque before and after bouts of load carriage and recovery (*n* = 14). Data represent mean ± SE; *p < 0.05 compared to pre value within the testing day; Percent changes represent difference from pre to post within the testing day; ^†^ *p* < 0.05 compared to pre value on BOUT1. *n* = 13 for isokinetic knee flexion and extension tests at 60 and 120°/s.

Multiple days of load carriage exercise had muscle group‐specific effects on isometric torque (Figure 2). No change was observed for the knee extensors, but there was a main effect of time on trunk extensor MVC (*p* = 0.0044; *η* ^2^ = 0.20), including within‐day changes following BOUT1 (−11%; *p* = 0.0311; *d* = 0.83) and BOUT2 (−12%; *p* = 0.0250; *d* = 0.86), but not BOUT3. Compared with BOUT1Pre, trunk extensor MVC was reduced at all subsequent time points (*p* range = 0.0002–0.0164), except for BOUT2Pre.

Similar to the pattern observed for isometric torque, changes in isokinetic torque varied by muscle group, with the knee flexors more affected than the extensors. A main effect of time was noted for knee flexor MVC at 60°/s (*p* < 0.0001; *η* ^2^ = 0.34), with reductions at BOUT3Pre (−12%; *p* = 0.0403; *d* = 1.20), BOUT3Post (−16%; *p* = 0.0007; *d* = 1.68) and RECPost (−17%; *p* = 0.0002; *d* = 1.84) compared with BOUT1Pre, and no change in knee extensor MVC. Likewise, no changes in knee extensor MVC at 120°/s were detected across load carriage bouts, but knee flexor MVC was lower (*p* = 0.0069; *η* ^2^ = 0.19) at both BOUT3Post (−12%; *p* = 0.0435; *d* = 1.19) and RECPost (−12%; *p* = 0.0466; *d* = 1.21) relative to BOUT1Pre.

Changes in isokinetic torque of the trunk extensors and flexors were observed at the slower angular velocity (60°/s). There was a main effect of time on trunk extensor MVC at 60°/s (*p* = 0.0155; *η* ^2^ = 0.18) as BOUT2Post (−14%, *p* = 0.0451; *d* = 1.23), BOUT3Post (−15%; *p* = 0.0137; *d* = 1.39) and RECPost (−14%; *p* = 0.0265; *d* = 1.31) were all lower than BOUT1Pre. In contrast, no changes in trunk flexor MVC at 60°/s were evident across load carriage bouts. Finally, there was a main effect of time on trunk flexor MVC at 120°/s (*p* = 0.0211; *η* ^2^ = 0.17), but no pairwise differences were observed.

### 3.4 Systemic inflammatory response

**FIGURE 3:**
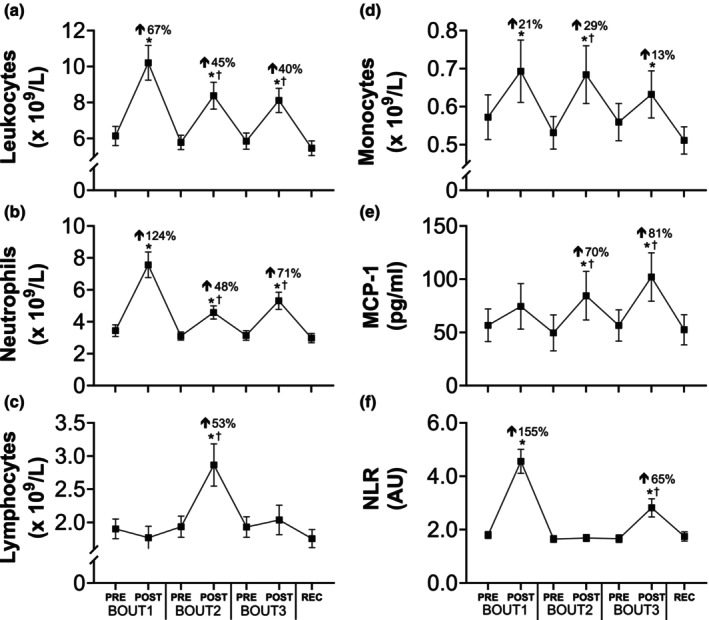
Systemic inflammation in response to repeated bouts of load carriage exercise. Total circulating leukocyte (a), neutrophil (b), lymphocyte (c), monocyte (d), and monocyte chemoattractant protein‐1 (MCP‐1; e) concentrations, and neutrophil‐to‐lymphocyte ratio (NLR; f) before and after bouts of load carriage and recovery (*n* = 14). Data represent mean ± SE; *p < 0.05 compared to pre value within testing day; Percent changes represent the difference from pre to post within testing day; ^†^ *p* < 0.05 compared to pre value on BOUT1. *n* = 9 for MCP‐1.

There was a main effect of time on total circulating leukocytes (*p* < 0.0001; *η* ^2^ = 0.35), neutrophils (*p* < 0.0001; *η* ^2^ = 0.47), and monocytes (*p* < 0.0001; *η* ^2^ = 0.09), with each increasing following every load carriage bout (Figure 3). While the load carriage tasks were identical on BOUT1 and BOUT3 and elicited similar HR responses, the change in total leukocyte (Δ = 4.07 vs. 2.27 x 10^9^/L; *p* = 0.0046) and neutrophil (Δ = 4.12 vs. 2.17 x 10^9^/L; *p* = 0.0038) counts was attenuated following the latter bout. The neutrophil‐to‐lymphocyte ratio (NLR) followed a similar pattern as neutrophils, whereby the increase on BOUT3 was attenuated >2‐fold relative to BOUT1 (Δ = 2.76 vs. 1.16 AU; *p* = 0.0021). The NLR did not change following BOUT2 due to commensurate increases in neutrophil and lymphocyte cell counts. In contrast, the greatest changes in lymphocytes (+53%; *p* < 0.0001; *d* = 2.59) and monocytes (+29%; *p* = 0.0016; *d* = 1.57) occurred following the 4‐mile time trial on BOUT2.

In a subset of participants (*n* = 9), we also examined serum MCP‐1 concentrations to characterize the chemokine response to our protocol as it regulates myeloid cell chemotaxis (Zahorec, 2021). There was a main effect of time on MCP‐1 concentration (*p* < 0.0001; *η* ^2^ = 0.11), including within‐day changes on BOUT2 (+70%; *p* = 0.0233; *d* = 1.59) and BOUT3 (+81%; *p* = 0.0011; *d* = 2.07), in contrast with attenuated leukocyte and neutrophil responses during subsequent exercise bouts.

### 3.5 Biochemical markers of muscle damage

**FIGURE 4:**
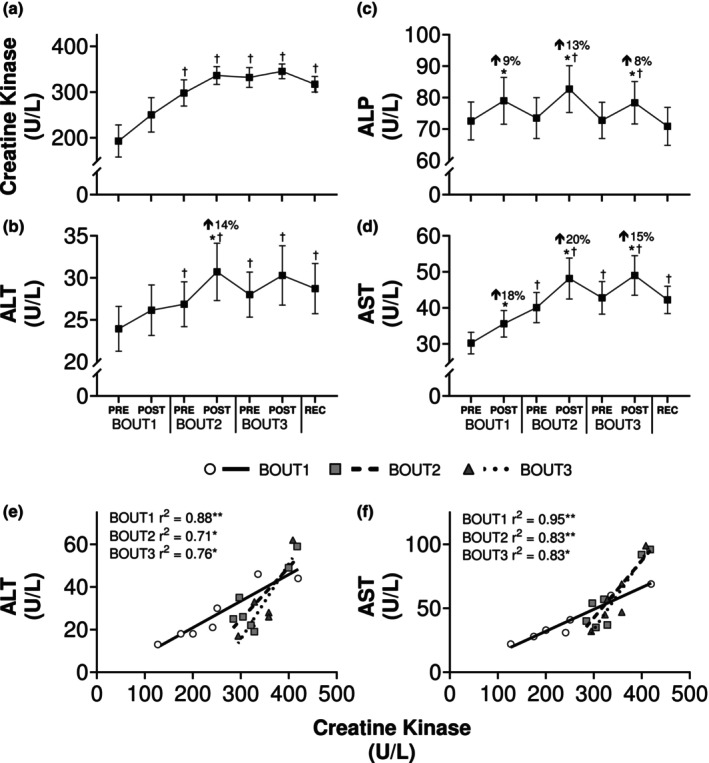
Biochemical markers of muscle damage in response to repeated bouts of load carriage exercise. Circulating creatine kinase (a), alanine transaminase (ALT; b), alkaline phosphatase (ALP; c), and aspartate aminotransferase (AST; d) before and after bouts of load carriage and recovery (*n* = 14). Data represent mean ± SE; *p < 0.05 compared to pre value within testing day; Percent changes represent differences from pre to post within testing day; ^†^ *p* < 0.05 compared to pre value on BOUT1. Bottom (e–f): Scatterplots showing relationships between creatine kinase and ALT (e) and AST (f) concentrations following each bout of load carriage exercise. *p < 0.05; **p < 0.01. *n* = 7 and 6 for creatine kinase on BOUT1/2 and BOUT3, respectively.

Serum creatine kinase was 192.7 ± 35.1 U/L at baseline, falling within the normal range of values for healthy adult men (Centers for Disease Control and Prevention (CDC), 2011). There was a main effect of time on creatine kinase (*p* < 0.0001; *η* ^2^ = 0.39), which was increased at BOUT2Pre and remained elevated for the duration of the protocol (Figure 4a). Main effects of time were also noted for serum ALT (*p* < 0.0001; *η* ^2^ = 0.04), ALP (*p* < 0.0001; *η* ^2^ = 0.03), and AST (*p* < 0.0001; *η* ^2^ = 0.13), although the only within‐day change for ALT was on BOUT2 (Figure 4c,d). While ALP returned to baseline each morning, elevated levels of ALT and AST persisted throughout the study. For comparison with other studies, we examined relationships between these liver enzymes and creatine kinase. At the post timepoint following all bouts of load carriage, creatine kinase had a strong and positive association with ALT (BOUT1: *r* = 0.94, *p* = 0.0025; BOUT2: *r* = 0.84, *p* = 0.0020; BOUT3: *r* = 0.87, *p* = 0.0053) and AST (BOUT1: *r* = 0.97, *p* < 0.0001; BOUT2: *r* = 0.91, *p* < 0.0001; BOUT3: *r* = 0.91, *p* < 0.0001) (Figure 4e,f), while no relationships were evident with ALP.

### 3.6 Red blood cell parameters

**FIGURE 5:**
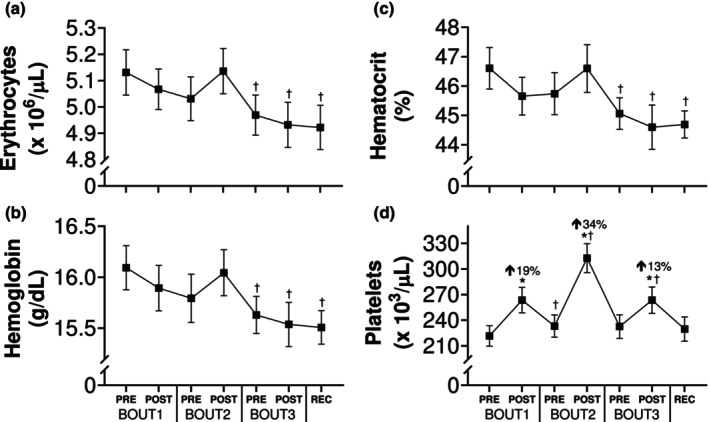
Red blood cell indices in response to repeated bouts of load carriage exercise. Erythrocyte (a), hemoglobin (b), hematocrit (c), and platelet (d) values before and after bouts of load carriage and recovery (*n* = 14). Data represent mean ± SE; *p < 0.05 compared to pre value within testing day; Percent changes represent difference from pre to post within testing day; ^†^ *p* < 0.05 compared to pre value on BOUT1.

Main effects of time were noted for erythrocytes (*p* = 0.0004; *η* ^2^ = 0.07), hemoglobin (*p* = 0.002; *η* ^2^ = 0.08), and hematocrit (*p* = 0.0007; *η* ^2^ = 0.09), while no within‐day changes were obvious for any parameter (Figure 5). However, hemoglobin and hematocrit were reduced by ~4% after BOUT3 and remained lower through the recovery visit, compared with BOUT1Pre. Platelet levels also increased (*p* < 0.0001; *η* ^2^ = 0.24) following each bout of load carriage, with the greatest change (+34%) occurring after the 4‐mile time trial on BOUT2.

### 3.7 Muscle pain, soreness, and discomfort

As load carriage exercise involves multi‐joint movements and several large muscle groups, we evaluated the presence of soreness throughout the anterior and posterior chains. Compared with the baseline value (BOUT1Pre), participants reported increased soreness for the posterior deltoids (*Х* ^2^ = 18.570; *p* = 0.0096) and upper trapezius (*Х* ^2^ = 14.325; *p* = 0.0497) at the post timepoint for BOUT1, BOUT2, and BOUT3. In contrast, no change was detected in any other muscle group, indicating soreness was confined to muscles of the upper posterior chain.

### 3.8 Relationships between immune cell counts and skeletal muscle function

**FIGURE 6:**
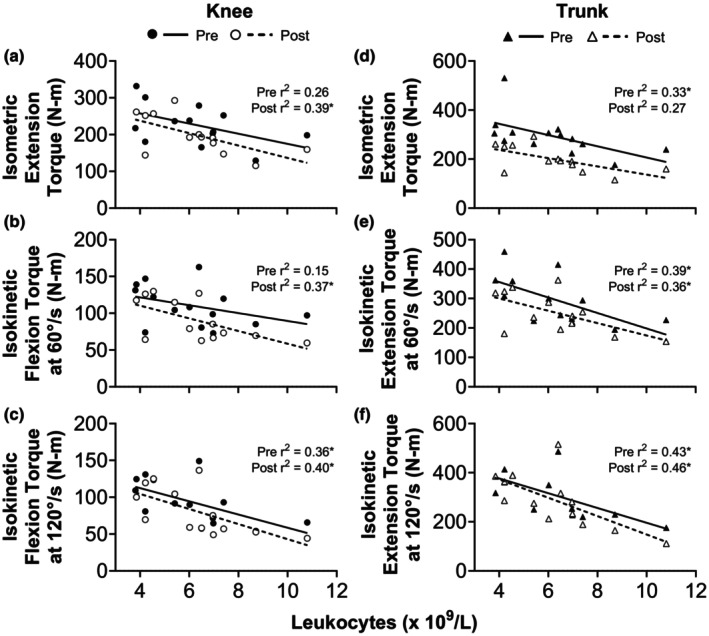
Relationships between immune status and skeletal muscle function at baseline and recovery. Scatterplots showing the relationship between total circulating leukocyte count and isometric and isokinetic torque production for knee flexion (a–c) and trunk extension (d–f) at baseline/pre (BOUT1Pre) and recovery/post (RECPost). *p < 0.05.

At baseline (BOUT1Pre), total leukocyte count was negatively associated with isokinetic knee flexion MVC at 120°/s (*r* = −0.60, *p* = 0.0228) as well as trunk extension MVC under isometric (*r* = −0.57, *p* = 0.0318) and isokinetic (60°/s: *r* = −0.63, *p* = 0.0223; 120°/s: *r* = −0.66, *p* = 0.0148) conditions (Figure 6). Greater leukocyte counts at baseline also predicted reduced MVC during recovery (RECPost) for isometric knee extension (*r* = −0.63, *p* = 0.0216), isokinetic knee flexion (60°/s: *r* = −0.61, *p* = 0.0272; 120°/s: *r* = −0.63, *p* = 0.0211), and isokinetic trunk extension (60°/s: *r* = −0.60, *p* = 0.0301; 120°/s: *r* = −0.68, *p* = 0.0111).

## 4 DISCUSSION

Load carriage is a common and essential mode of physical activity for military personnel and has physiological and biomechanical consequences, including changes to the metabolic cost of walking (Huang & Kuo, 2014), gait mechanics (Walsh & Low, 2021), and markers of bone formation (Staab et al., 2023). This study examined the effects of repeated load carriage bouts, which varied in external load, duration, and intensity, on indirect markers of EIMD, liver function tests, and red blood cell parameters in male soldiers. There were multiple changes consistent with EIMD, including reduced muscle strength and systemic inflammation, as well as altered liver enzymes and oxygen‐carrying capacity. In addition, greater leukocyte counts at baseline predicted lower knee and trunk strength during the recovery period. Collectively, our findings suggest that multiple days of heavy load carriage in highly trained infantry soldiers result in mild EIMD and alterations to oxygen‐carrying capacity, which, without adequate recovery, could impair physical performance (Boffey et al., 2019) and/or heighten musculoskeletal injury risk (Gill et al., 2023).

Weakness is a hallmark of skeletal muscle tissue damage and has been suggested as the best indirect marker of EIMD (Damas et al., 2016). The notion that load carriage can cause EIMD is supported by previous research showing reduced knee extensor torque (10%–15%) after a single bout of loaded walking (Blacker et al., 2010, 2013; Grenier et al., 2012), which is exacerbated following a subsequent bout (James et al., 2021). Those studies, however, involved recreationally active young adults rather than soldiers accustomed to load carriage. Here, we found load carriage caused changes in MVC strength that were largely cumulative and muscle group‐specific. For instance, isometric knee extensor torque was maintained throughout the protocol, in contrast with prior work (Blacker et al., 2010, 2013; James et al., 2021), but trunk extensor torque was reduced at nearly every timepoint relative to baseline (Figure 2). The reduced torque we observed likely reflects EIMD, given the delayed onset (2–3 d) and prolonged duration (≥24 h) of most strength decrements (Stozer et al., 2020). Local skeletal muscle fatigue due to central and peripheral mechanisms (Blacker et al., 2013) is an alternative explanation for the loss of strength immediately post‐exercise, but it is unlikely as fatigue‐induced declines in torque are rapidly (e.g., within 10 min) restored in young adults after a series of repeated dynamic contractions (Kent‐Braun et al., 2014). Intense military training exercises can also reduce strength through weight loss due to a net negative energy balance (Murphy et al., 2018). However, body weight remained stable in the present study (e.g., −0.3 kg), meaning our finding of reduced torque cannot be explained by weight loss. Regardless, torque deficits that persist for ≥24 h after load carriage could compromise physical performance during multiday operations. Lower strength may impair readiness for essential tasks (Nindl et al., 2015) and heighten musculoskeletal injury risk, especially with 70% of all injuries among active‐duty soldiers attributable to overuse (Molloy et al., 2020). Overall, maximal muscle strength was 15%–20% lower after three bouts of load carriage, meaning physical performance could be reduced, even in highly trained soldiers, during subsequent activities until adequate recovery time is provided.

Systemic inflammation is a cardinal sign of EIMD (Clarkson & Hubal, 2002), and the magnitude is dependent upon the mode, intensity, and duration of physical activity (Freidenreich & Volek, 2012; Nieman & Wentz, 2019). Currently, knowledge of the inflammatory response to load carriage is informed by only two studies (Jensen et al., 2019; Pasiakos et al., 2016), both of which measured a single cytokine following an acute bout of load carriage. Our work builds upon these studies by examining the systemic immune response, including leukocyte subtypes and a primary chemokine (MCP‐1) involved in myeloid cell chemotaxis, to multiple load carriage bouts. We found that each bout of load carriage significantly elevated total leukocyte, neutrophil, and monocyte counts (Figure 3). In comparison to unloaded walking at a similar intensity (Nieman et al., 2005), our results show a more pronounced immune response, with larger increases in total leukocyte (+67% vs. +24%) and neutrophil (+124% vs. +21%) counts after one bout. This suggests the greater immune response we observed is likely due to the mechanical strain placed upon skeletal muscle fibers, leading to disrupted sarcomeres and tissue damage (Proske & Morgan, 2001). The infiltration of myeloid cells, including neutrophils and monocytes, into injured tissue is a critical component of the acute inflammatory response, facilitating the processes of remodeling and repair (Tidball & Villalta, 2010). Our findings align with previous research on both isolated muscle groups (Ebbeling & Clarkson, 1990; Heckel et al., 2019; Hirose et al., 2004; Pizza et al., 2001) and whole‐body exercises, such as marathon running (Kratz et al., 2002). Coupled with the reduction in muscle strength, we interpret the inflammatory response to reflect mild EIMD following load carriage exercise.

How load carriage mode and intensity affect leukocyte subfractions is further apparent through the neutrophil‐to‐lymphocyte ratio (NLR), a marker that reflects the balance between innate (neutrophils) and adaptive (lymphocytes) immune responses (Zahorec, 2021). In our study, NLR increased after the loaded walks on BOUT1 (+155%) and BOUT3 (+65%), but not after the 4‐mile run/walk on BOUT2, due to commensurate increases in neutrophils and lymphocytes. Our finding that lymphocyte levels increased after the loaded run, but not walks, is consistent with previous research on aerobic exercise intensity (Shek et al., 1995). Acute aerobic exercise increases blood flow, resulting in shear stress, and also stimulates the secretion of catecholamines, both of which are hypothesized to mediate the release of lymphocytes into circulation (Walsh et al., 2011). The lower aerobic intensity during the loaded walks may not have initiated these mechanisms, in contrast with the 4‐mile run, and this may explain the differential lymphocyte responses.

Migration and trafficking of circulating immune cells to peripheral sites of tissue damage is regulated by chemokines, or chemoattractant cytokines (Ramesh et al., 2013). MCP‐1 is a critical driver of monocyte chemotaxis (Takahashi et al., 2009), as well as other myeloid cells (Gschwandtner et al., 2019), and also promotes macrophage infiltration into injured skeletal muscle tissue (Lu et al., 2011). The acute effects of exercise on systemic MCP‐1 levels in the extant literature are equivocal, with some studies reporting an increase (Paulsen et al., 2005) and others a decrease (Ihalainen et al., 2014; Lagzdina et al., 2023). In our study, circulating MCP‐1 levels increased with each bout of activity, including changes after the second (+70%) and third (+81%) bouts. In the presence of apparent EIMD, the amplified MCP‐1 response may be an attempt to facilitate healing by augmenting the pool of tissue‐resident macrophages in skeletal muscle (Deyhle et al., 2015), possibly toward a more anti‐inflammatory subtype for repair, and/or by driving myoblast proliferation, a mechanism that is independent of leukocytes (Yahiaoui et al., 2008). However, additional work is needed to determine the MCP‐1 response to EIMD, both systemically (blood) and locally (muscle).

The temporal profile of the immune response to repeated bouts of exercise has been investigated following rowing (Nielsen et al., 1996), cycling (Nieman et al., 2007), and running (Mackinnon & Hooper, 1994), with mixed findings depending on leukocyte subfractions. Our study adds to this body of literature by reporting changes in immune cell counts during three successive bouts of load carriage, including two different modes and intensities (walking with 50% body weight at ~60% HR_max_ vs. running with 30% body weight at ~80% HR_max_). We found attenuated leukocyte and neutrophil responses after a second, and identical, bout of load carriage 48 h later (Figure 3), similar to the repeated bout effect (McHugh, 2003). However, the repeated bout effect is classically indicated by partial or full preservation of muscle strength, which we did not observe, suggesting the initial bout did not afford protection against future damage. Despite this, altered inflammation is hypothesized to be particularly important during the early stages (~1 d) of adaptation to strenuous and/or unaccustomed activity (Hyldahl et al., 2017). Our findings at the systemic level (attenuated neutrophil counts but enhanced MCP‐1 response) agree with preclinical work showing reduced intramuscular neutrophil infiltration (Paulsen et al., 2010) but upregulated MCP‐1 gene expression (Hubal et al., 2008), following a second bout of exercise, suggesting there may be complex and reciprocal changes among leukocytes and cytokines working in concert to repair skeletal muscle following EIMD. Future research involving other groups of well‐trained individuals (e.g., athletes and military) are needed to confirm these findings.

The presence of intramyocellular enzymes in the blood provides indirect evidence of EIMD as these proteins leak into circulation when muscle fibers are ruptured (Kanda et al., 2014). ALT and AST are considered clinical indicators of liver function (Iluz‐Freundlich et al., 2020); however, isoforms of these transaminases are also present within skeletal muscle tissue and can increase due to extrahepatic causes (Janssen et al., 1989). We found that each bout of load carriage increased serum levels of ALT, AST, and ALP, with ALT and AST remaining elevated during recovery (Figure 4). This is consistent with previous research showing elevated liver function tests persist for several days following a single bout of muscle‐damaging activity, such as unaccustomed resistance training (Pettersson et al., 2008) or a marathon (Niemela et al., 2016). And because we tested soldiers regularly engaged in moderate‐ to vigorous‐intensity exercise, these responses would likely be more pronounced in untrained individuals. We also found a strong positive association between postexercise levels of ALT and AST and creatine kinase, an indirect marker of muscle tissue damage (Baird et al., 2012). A relationship between these liver enzymes and creatine kinase has been observed in rhabdomyolysis (Lim et al., 2019), but our data suggest this relationship exists in the absence of pathology, meaning liver function tests may serve as a proxy for subclinical muscle tissue damage in highly‐trained individuals.

We found that multiple bouts of loaded walking reduced circulating erythrocyte levels and, in turn, elicited a ~4% decrease in hemoglobin and hematocrit by the end of the protocol (Figure 5). To our knowledge, this is the first study to show that load carriage results in exercise‐induced hemolysis, or the rupture and destruction of erythrocytes in circulation with physical activity (Lippi & Sanchis‐Gomar, 2019). Although less than prolonged endurance events (Kratz et al., 2002; Wu et al., 2004), this change could have implications for aerobic performance as a 1.9% reduction in hematocrit is equivalent to a 1‐unit (300 cc packed red blood cells) loss of blood (Elzik et al., 2006). Several mechanisms are thought to play a role in exercise‐induced hemolysis (Senturk et al., 2005; Yusof et al., 2007), including capillary compression associated with footstrike (Telford et al., 2003), which may be particularly impactful in the presence of a heavy external load. Our findings suggest that 3 days of load carriage tasks could negatively impact endurance performance via reduced oxygen‐carrying capacity, which, combined with decreased skeletal muscle function noted above, could further impair physical performance during prolonged training or missions.

An inverse relationship between leukocyte counts and skeletal muscle function has recently been reported among diverse populations, from adolescents (Lopez‐Gil et al., 2021) to active‐duty military (Chung et al., 2020) and older adults (Tuttle et al., 2020). Our findings lend support to this notion, as greater leukocyte levels were associated with lower knee flexor and trunk extensor MVC at baseline and recovery (Figure 6). Whether this relationship is bidirectional remains unclear, but circulating leukocytes could impair muscle mass and/or strength via the secretion of pro‐inflammatory cytokines. In cell culture and animal models, exposing myoblasts to cytokines inhibits markers of myogenesis (Howard et al., 2020). In contrast, other studies show that circulating leukocyte counts are associated with clinical endpoints independent of cytokines (Leng et al., 2007; Willems et al., 2010). While the mechanisms require further study, our results and others (Chung et al., 2020; Lopez‐Gil et al., 2021; Tuttle et al., 2020) suggest immune cell counts could help identify individuals with lower skeletal muscle strength, a target for ongoing efforts to mitigate musculoskeletal injury risk among soldiers (Hughes et al., 2019).

This study has some limitations that should be acknowledged. First, our experimental design lacks a control group (e.g., unloaded walking) for comparison with the loaded walking condition. However, unloaded walking has minimal effects on muscle strength (Blacker et al., 2013) and the immune response (Nieman et al., 2005), meaning changes we observed likely reflect the heavy external loads. We also had a reduced sample size for creatine kinase (*n* = 7) and MCP‐1 (*n* = 9) due to logistical challenges with the coronavirus pandemic (COVID‐19). Nonetheless, we observed a significant main effect of time on both outcomes, indicating adequate statistical power. Finally, only male soldiers completed this study, which is a limitation given the importance of sex as a biological variable in research (Arnegard et al., 2020). As sex‐based differences in physiological responses to military field training exercises have been reported (Vikmoen et al., 2020), future work should include both male and female soldiers to determine if there are sex‐specific adaptations to load carriage tasks.

In conclusion, repeated bouts of load carriage resulted in symptoms consistent with EIMD, including systemic inflammation and decreases in knee and trunk strength, elevated liver enzyme levels, and reduced oxygen‐carrying capacity in well‐trained soldiers accustomed to vigorous activity. Collectively, these changes could compromise readiness and/or predispose soldiers to injury, which highlights the need for practical strategies (e.g., nutraceuticals, exercise training, and adequate recovery) to limit EIMD and optimize physical performance. Future studies are needed that test novel approaches to mitigating the physiological effects of load carriage on male and female soldiers, with the ultimate goal of enhancing readiness for military operations.

## AUTHOR CONTRIBUTIONS

K.L.M. and K.S.O. conceived and designed research; C.R.S., K.L.M., A.L.S., K.R., A.H.M., T.N., and K.S.O. collected data; C.R.S. and K.S.O. analyzed data, interpreted data, and drafted the manuscript; C.R.S., K.L.M., A.L.S., K.R., A.H.M., T.N., and K.S.O. edited and revised the manuscript and approved the final version of the manuscript.

## FUNDING INFORMATION

This work was supported by the U.S. Army DEVCOM Soldier Center under the Measuring and Advancing Soldier Tactical Readiness and Effectiveness (MASTR‐E) program.

## CONFLICT OF INTEREST STATEMENT

No conflicts of interest, financial or otherwise, are declared by the authors.

## DISCLAIMER

The opinions or assertions contained herein are the private views of the authors and are not to be construed as official or as reflecting the views of the Army or the Department of Defense. Any citations of commercial organizations and trade names in this report do not constitute an official Department of the Army endorsement or approval of the products or services of these organizations. Approved for public release; distribution is unlimited.

## ETHICS STATEMENT

All individuals provided written informed consent prior to participation, and all procedures within this protocol were approved by the Institutional Review Board at the U.S. Army Combat Capabilities Development Command Armaments Center (Picatinny, NJ, United States).

## Data Availability

Study data were collected as part of the ongoing U.S. Army DEVCOM Soldier Center MASTR‐E program and are not publicly available at this time.
